# ASSOCIATIONS OF GENETICS AND LIFE COURSE CIRCUMSTANCES WITH A NOVEL AGING MEASURE THAT CAPTURES MORTALITY RISK

**DOI:** 10.1093/geroni/igz038.1177

**Published:** 2019-11-08

**Authors:** Zuyun Liu, xi Chen, Thomas M Gill, Chao Ma, Eileen Crimmins, Morgan E Levine

**Affiliations:** 1 Department of Pathology, Yale School of Medicine, New Haven, Connecticut, United States; 2 Department of Health Policy and Management, Yale School of public Health Department of Economics Yale University, New Haven, Connecticut, United States; 3 Yale School of Medicine, New Haven, Connecticut, United States; 4 Davis School of Gerontology, University of Southern California, Los Angeles, California, United States

## Abstract

We aimed to evaluate associations between a comprehensive set of factors, including genetics and childhood and adulthood circumstances, and a novel aging measure, Phenotypic Age (PhenoAge), which has been shown to capture mortality and morbidity risk in the U.S. population. Using data from 2339 adults (aged 51+) from the U.S. Health and Retirement Study, we found that together all 11 study domains (4 childhood and adulthood circumstances domains, 5 polygenic scores [PGSs] domains, and 1 demographics, and 1 behaviors domains) accounted for about 30% of variance in PhenoAge after accounting for chronological age. Among the 4 circumstances domains, adulthood adversity was the largest contributor (9%), while adulthood socioeconomic status (SES), childhood adversity, and childhood SES accounted for 2.8%, 2.1%, 0.7%, respectively. All PGSs contributed 3.8% of variance in PhenoAge (after accounting for chronological age). Further, using Hierarchical Clustering, we identified 6 distinct subpopulations/clusters based on the 4 circumstances domains, and 3 subpopulations/clusters of them that appear to represent disadvantaged circumstances were associated with higher PhenoAge. Finally, there was a significant gene-by-environment interaction between a previously validated PGS for coronary artery disease and the most apparently disadvantaged subpopulation/cluster, suggesting a multiplicative effect of adverse life course circumstances coupled with genetic risk on phenotypic aging. We concluded that socioenvironmental circumstances during childhood and adulthood account for a sizable proportion of differences in phenotypic aging among U.S. older adults. The disadvantaged subpopulations exhibited accelerated aging and the detrimental effects may be further exacerbated among persons with genetic predisposition to coronary artery disease.

